# Understanding Mechanism of Photocatalytic Microbial Decontamination of Environmental Wastewater

**DOI:** 10.3389/fchem.2018.00033

**Published:** 2018-02-28

**Authors:** Chhabilal Regmi, Bhupendra Joshi, Schindra K. Ray, Gobinda Gyawali, Ramesh P. Pandey

**Affiliations:** ^1^Department of Environmental and Bio-chemical Engineering, Sun Moon University, Asan-si, South Korea; ^2^Department of Life Science and Bio-chemical Engineering, Sun Moon University, Asan-si, South Korea; ^3^Department of BT-Convergent Pharmaceutical Engineering, Sun Moon University, Asan-si, South Korea

**Keywords:** photocatalysis for environment, microbial inactivation, mechanisms of action, semiconductors, environmental pollutants

## Abstract

Several photocatalytic nanoparticles are synthesized and studied for potential application for the degradation of organic and biological wastes. Although these materials degrade organic compounds by advance oxidation process, the exact mechanisms of microbial decontamination remains partially known. Understanding the real mechanisms of these materials for microbial cell death and growth inhibition helps to fabricate more efficient semiconductor photocatalyst for large-scale decontamination of environmental wastewater or industries and hospitals/biomedical labs generating highly pathogenic bacteria and toxic molecules containing liquid waste by designing a reactor. Recent studies on microbial decontamination by photocatalytic nanoparticles and their possible mechanisms of action is highlighted with examples in this mini review.

The natural ecosystem is being deteriorated continuously because of unrestrained flow of wastewater from various industries, hospital wastes, and laboratories due to rapid and poorly-planned urbanization in developing countries and development of mega cities. The urban population is rising unprecedentedly. The socio-economic disparities is exacerbating while creating unsanitary conditions that facilitates the outbreaks. The natural water bodies are contaminated with (i) pathogens originated from hospitals and biomedical laboratories, (ii) different hazardous materials such as organic dyes, chemicals, and toxins from industries. Meanwhile, fertile agricultural lands are also affected with those chemicals and excessively used hazardous pesticides, herbicides, and plastics while unduly used fertilizers are causing algal blooms in natural water bodies (Rosen et al., 2017; West et al., 2017). Ultimately, these harmful chemicals are entering to humans to cause deadly diseases *via* food chain from plants and animals. In some instances, the contaminated water and foods caused severe epidemics and pandemics due to super-bacteria resistant to almost all drugs. In another hand, the genetic disorders caused by chemical mutagens leaving thousands of people with deadly non-transmissible diseases such as cancers, reproductive disorders and adverse pregnancy outcomes, physical disorders, and disabilities (Frazier and Hage, 1998). All these factors are amplifying the global risks (www.weforum.org). Thus, the environment protection is always crucial to secure the human life and animals on the planet. Though governments of different nations issued standard guidelines to discharge treated effluents, sewerage, and other wastages to the environment, yet excessive discharge of waste is maltreating the environment and the ecosystem. Hence, there is an urgent need for a highly efficient system to treat waste for complete degradation of toxic molecules into simpler non-toxic forms while inactivating highly pathogenic multidrug-resistant superbugs to make water free of pathogens.

Many industries and laboratories treat chemical wastages using various approaches prior to discharge them. Commonly practiced approaches include- (1) physical methods- sedimentation, floatation, filtration; (2) biological oxidation methods- includes trickling filters, rotating biological contractor, activated sludge, lagoons, and various aerobic and anaerobic methods; and (3) chemical treatment methods such as coagulation and flocculation by lime added with ferric chloride and lime added with aluminum sulfate. Hospitals and biomedical laboratories treat microbially contaminated wastes by either physical means such as autoclaving and irradiation, or chemical treatment methods using organic solvents and detergents to remove or inactivate pathogens prior to discharge. All these methods have their own merits and demerits. The major demerit lies in the incomplete removal of these wastage products as well as the formation of intermediates which are more toxic than the parent pollutants.

Recently, photocatalysis, a novel green technology which is based on advance oxidation processes (AOPs') and is capable of generating different reactive species such as reactive oxygen species (ROS), reactive nitrogen species (RNS), oxygen radicals (·${\text{O}}_{2}^{-}$), hydroxyl radicals (·OH) have attracted the great attention for their potential application for the degradation of organic and biological wastes (Cavassin et al., 2015; Ramazanzadeh et al., 2015; Waiskopf et al., 2016; Nosaka and Nosaka, 2017; Podporska-Carroll et al., 2017). In this method, the synthesized semiconductor NPs are activated by light of different wavelength (1) UV light, (2) visible light, and (3) NIR light depending on the energy band gap they possess (Sarina et al., 2013). The activated semiconductor degrade the organic and microbial cells by a simple photocatalytic redox mechanism. In recent year, extensive research has been carried out for the efficient removal of the toxic molecules and pathogenic microbes from wastewater using semiconductor photocatalytic NPs. Moreover, various modification on the photocatalytic nanoparticles such as formation of heterojunction, tuning the morphology, doping of metals/non-metals, and decorating of nanoparticles onto the photocatalytic surface have been carried out in order to make efficient inactivation of microorganism in waste water under visible light. Regmi et al. (2017a) have fabricated Fe doped BiVO_4_ visible light active semiconductor and showed efficient removal of ibuprofen and *Escherichia coli* in water. Recently, the synthesis of Bi_2_MoO_6_ on g-C_3_N_4_ nanosheet is reported with enhanced photocatalytic hydrogen evolution and disinfection of *E. coli* under the visible light (Li et al., 2017). Meanwhile, Meng et al. (2017) used synthesized MoS_2_ quantum dots interspersed Bi_2_WO_6_ heterostructure for efficient detoxification and disinfection in wastewater. Doping of different size of metals such as Ag further improved the photocatalytic antibacterial activities (Esfandiari et al., 2014). Ning et al. (2017) synthesized dual couples Bi metal depositing and Ag@AgI islanding on BiOI 3D architectures showed synergistic bactericidal mechanism of *E. coli* under visible light. Similarly, Booshehri et al. (2014) studied the effect of depositing silver nanoparticles on BiVO_4_ to enhance visible light photocatalytic inactivation of bacteria in water. Other illustrations of microbial inactivation using semiconductors include the heterogeneous photocatalytic inactivation of *B. stearothermophilus* endospores in aqueous suspensions under artificial and solar irradiation (Berberidou et al., 2012), visible light inactivation of bacteria and fungi by modified titanium dioxide (Mitoraj et al., 2007), and the efficient inactivation of *E. coli* by ZnO–Ag nanoparticles under solar radiation (Adhikari et al., 2015). Similarly, visible light driven morphology tuned α-NiMoO_4_ photocatalyst were used to inactivate multidrug resistant *Staphylococcus aureus* (Ray et al., 2018). Diverse microbes such as pathogenic bacteria, viruses, fungi, protozoa, and other organisms are inactivated thus making the water devoid of microbes and safe to human, animal, and environment.

Another key importance of semiconductor NPs is its multiple applications in medicine, bio-imaging, drugs delivery, the coating of implantable devices, wound dressings, dental material, bone cement as well as personal care products (Smith and Nie, 2010; Pan et al., 2016; Wang et al., 2017). They can also prevent pathogenic microbes to restrict the biofilm formation. Biofilm-forming bacteria such as *S. aureus* is one of the deadliest superbugs which is getting resistant to almost all kinds of drugs. NPs such as MgF_2_, Au-based NPs (Yu et al., 2016), AgBr-Ag-Bi_2_WO_6_ (Zhang et al., 2010), Ag and Mg-based NPs (Lellouche et al., 2012; Markowska et al., 2013), ZnO, CuO, Fe_3_O_4_, TiO_2_ and YF photocatalytic NPs are capable of inhibiting such microbes from developing biofilm (Wang et al., 2017).

Harmful algal bloom is another major problem in natural water bodies where the dissolved oxygen is extremely low, killing marine lives. Some of these algae produce toxins, algal toxins and metabolites such as domoic acid, brevotoxin, saxitoxin, microcystin, geosmin, anabena, nodularin etc. which are also detrimental to the health of the marine animals (Sivonen and Jones, 1999). As these harmful algae last for several months to years in the water bodies, these dead zones lost all forms of plants and marine animals while causing significant loss of environmental health, ecosystem, and economies. Thus, photocatalytic materials could play a potential role in decreasing such harmful algal blooms and preventing natural waters and different form of lives and ecosystem. The Ni-doped BiVO_4_ photocatalyst exhibited the efficient algae inactivation under visible light irradiation while Co-BiVO4 degraded malachite green and inactivated harmful microorganisms in wastewater including green tides (Regmi et al., 2017b,c). Similarly, Ag-TiO_2_ has shown efficient UV-photo elimination of harmful algal bloom (Rodríguez-González et al., 2010).

The multiple applications of photocatalytic NPs lead to discovering the modes of action of these particles in decomposing organic molecules to simpler form such as CO_2_ and H_2_O and disabling actively growing pathogens from multiplying and reproducing. This potential of NPs has huge benefits to make the environment clean by eliminating toxic molecules and pathogens while keeping the entire ecosystem healthy.

The mechanisms of destruction of the microorganisms, algae, and various organic toxic molecules is different. Although several studies have been reported till now to explain the possible mechanism of degradation of organic compounds and killing of microbes, the antimicrobial mechanism of photocatalytic NPs has to be explored yet. The photocatalytic process which is based on AOPs' is considered as green technique for treatment of polluted water containing harmful microbes. In this process a semiconductor upon absorption of a photon of suitable energy (≥band gap energy) can act as a photocatalytic substrate by producing highly reactive radicals that can that can degrade indiscriminately micropollutants including harmful micro-organism present in wastewater (Pillai et al., 2017). But during the process majorities of the produced charges undergo recombination within the bulk/surface of photocatalyst, with the release of heat as a byproduct, before they are transferred to the adsorbate species creating the decrease in photocatalytic efficiency often greater than 90%. Thus, impurities substitution, lattice defects, and vacancies formation on the semiconductor can provide possible alternative pathways for the enhancement of photogenerated electrons and holes thus, prevent recombination.

The semiconductor photocatalyst on irradiation to light with energy equal or greater to its band gap energy produces an oxidative and reductive entity. In its first step, photo-generated holes and electrons are formed in the valence band (${\text{h}}_{\text{VB}}^{+}$) and the conduction band (${\text{e}}_{\text{CB}}^{-}$), respectively. Consequently, these photogenerated charge carriers then react with water or dissolved oxygen to produce reactive oxidizing species such as OH and ${\text{O}}_{2}^{-}$ that decompose pollutants into smaller molecules as well as inactivate micro-organisms.

$$\begin{array}{l} \text{Photocatalyst }+\text{ h}\nu \;\to \;{\text{e}}_{\text{CB}}^{-}\;+\;{\text{h}}_{\text{VB}}^{+}\\ {\text{h}}_{\text{VB}}^{+}\;+\;{\text{H}}_{2}\text{O }\to \;\cdot \text{OH }+\;{\text{H}}^{+}\\ {\text{h}}_{\text{VB}}^{+}\;+\;{\text{OH}}^{-}\;\to \;\cdot {\text{OH}}_{\text{ad}}\\ {\text{e}}_{\text{CB}}^{-}\;+\;{\text{O}}_{2}\;\to \;\cdot {\text{O}}_{2}^{-}\;\to^{2\text{H}+}\;{\text{H}}_{2}{\text{O}}_{2}\;\to^{\text{e}-}\;\cdot \text{OH }+\;{\text{OH}}^{-}\\ \cdot \text{OH }+\text{ pollutants/cellular constituent }(\text{micro}-\text{organism})\\ +\;{\text{O}}_{2}\;\to \text{ simpler products }(\text{salts},\;{\text{CO}}_{2},\;{\text{H}}_{2}\text{O etc}.,)\\ \cdot {\text{OH}}_{\text{ad}}\to \cdot {\text{OH}}_{\text{free}}+\text{pollutants }\to \text{ simpler oxidation products} \end{array}$$

Photocatalytic materials when come in contact with microbes via different energy such as electrostatic attraction, hydrophobic interactions, van der Waals forces and receptor-ligand interactions, they exert their effect on the bacterial cell membrane and starts to influence the basic metabolism of the cells by various mechanisms. The cells could also experience different stresses such as oxidative stresses, membrane permeability imbalance, changes in cell shape, protein inhibition and alteration in metabolism and DNA damages. As shown in Figure 1, various reactive radicals generated by photocatalytic NPs on receiving the photon energy higher than the band gap energy enters to the cells and brings disorders to the cells' metabolism and gene expression (Wang et al., 2015). Single NP can also have multiple modes of actions (Hajipour et al., 2012). Thus, the exact mechanism of cell death caused by NPs is currently unknown. Currently understood mechanisms of destruction of microbes are discussed with examples henceforward. (i) Oxidative stress induction; in which the excess ROS, generated as a result of redox process, favors the oxidation process in the cells that leads to the peroxidation of lipid membrane and eventually attack proteins, depress the activity of certain periplasmic enzyme, and eventually interact with DNA and damage it. (ii) Metal ion release; where the metal ions released from metal oxide semiconductors are percolated through the cell membrane and directly interact with the –SH, -NH and –COOH group of nucleic acid and protein and finally damaging them. However, this method is less lethal than the other. Since detail studies of metal ion release on antimicrobial mechanism has not been carried out yet; it is not considered as the major cause of cell death (Hussein-Al-Ali et al., 2014). (iii) The non-oxidative mechanism involves the inactivation of microbes by decreasing the critical cellular metabolism such as protein, amino acid, nucleotide, energy, and carbohydrate metabolism without oxidative stress induction. The mechanism of non-oxidative stress cell death is poorly understood with very few MgO NP (Leung et al., 2014). Among these three possible mechanisms of antibacterial activities of NPs, the first mechanism has attracted the most attention of the researchers.

**Figure 1:**
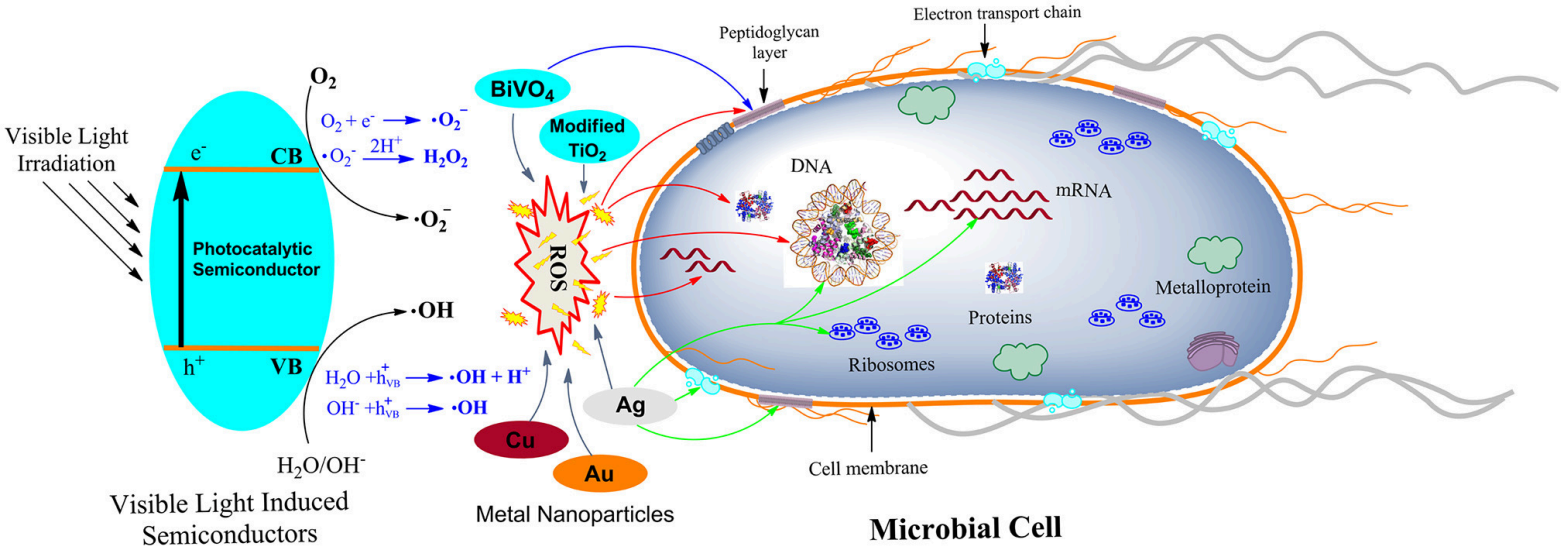
The possible mechanisms of antimicrobial activities exhibited by different photocatalytic semiconductors. In the left side of the figure, the activation of the photocatalytic semiconductor by visible light is shown. Red colored arrows point the targets of reactive oxygen species (ROS) generated by various semiconductors. The blue color arrow represents the target of BiVO_4_. Ag, Cu, and Au metal nanoparticles are also known to generate ROS and targets different parts in the cell. The green color arrow represents targets of Ag nanoparticle. Different targets in the microbial cells are labeled within the cell.

In general different types of ROS (·${\text{O}}_{2}^{-}$, ·OH, H_2_O_2_,) are generated by NPs by reducing O_2_ molecules (Figure 1). These ROS exert a different level of stress reactions to the cells to damage the cell components such as peptidoglycan layer, electron transport chain system, genomic materials (DNA, RNA), proteins, ribosomes etc. ROS alters the cell permeability by attacking the components of the cell membrane which changes cell integrity and release of cytoplasmic contents (Ansari et al., 2015; Huang et al., 2017; Ray et al., 2017). Moreover, ROS also depress the activities of various proteins essential for physiological processes in the cell (Padmavathy and Vijayaraghavan, 2011). H_2_O_2_ is able to enter the cell membrane and damage cell components while increasing the expression pattern of oxidative stress-induced genes (Xu et al., 2013).

The usefulness of photocatalysis for the disinfection of water has been shown by the destruction effects it has on a wide range of micro-organisms i.e. bacteria, viruses, fungi and protozoa (Laxma Reddy et al., 2017; Ray et al., 2017). The disinfection efficiency of photocatalysis depends upon pH, chemical nature of bacterial suspension medium, photocatalyst type, size, surface morphology, specific surface area, structure, zeta potential, high surface energy, and atomic ligand deficiency, concentration, light intensity, and treatment time as well as the nature of the targeted organism. The accepted sequence of events which take place when micro-organisms undergo photocatalytic experience are thought to be an attack of the cell wall constituent by the hydroxyl radical produced during the catalysis process. These radicals being the strongest agent could break many organic covalent bonds, such as C-C, C-H, C-N, C-O, and H-O present in biomolecules such as carbohydrates, nucleic acid, proteins, amino acid, and even DNA (Wu et al., 2016; Laxma Reddy et al., 2017; Xu et al., 2018).

Even though several modes of cell death based on reactive radicals are proposed and elucidated in recent publications, the exact mechanism of the antibacterial mechanism of NPs is not understood yet and warrants further research. Since there is variation in the type of NPs, strains, and environmental conditions used for antibacterial tests; it is very difficult to generalize the exact mechanism of killing of microbes by NPs. Different NPs exhibit different antibacterial activities over different strains. Thus, it is essential to bring such topics in the discussion forum for comprehensive analysis and elucidation of the exact mechanism of action of NPs. Several issues including entry of NPs to different cells having different structural integrity has to be elucidated. Moreover, the exact mechanism of NPs on protein degradation, DNA damage, and alteration of cells' basic metabolic pathways are hugely unknown.

Understanding the real mechanism of inhibiting and destroying cells during photocatalytic disinfection helps to fabricate more efficient semiconductor photocatalyst by (1) tuning the morphology, (2) incorporating transition metal ions such as Ag, Au, Cu, Fe, or Ni and (3) designing heterojunction photocatalysts capable of utilizing efficient energy source such as sunlight (Guo et al., 2015; Podporska-Carroll et al., 2017). Upon getting insights into the mechanism of the photocatalytic bactericidal phenomenon of semiconductor NPs, the system could be employed for large-scale decontamination of environmental wastewater or industries and hospitals/biomedical labs generating highly pathogenic bacteria and toxic molecules containing liquid waste by designing a reactor. Moreover, designing of semiconductor NPs which can utilize the large fraction of the solar spectrum (visible light) further expand the reactor's promiscuity without the expense of energy. More importantly, if these semiconductor NPs are reusable, non-toxic, and easily synthesizable; the waste treatment system will be highly cost-effective and sustainable. Another important point is that microbes need significant alteration to overcome the bactericidal activity exerted by NPs. NPs act on wide target sites in the cell at a time, thus bacteria could not make such big changes in metabolism by making mutational changes in the genomes and bringing changes in the metabolism in short period of time. This eliminates the concern of pathogens getting resistance to such treatment systems. Not only wastewaters, such reactors can also be designed for different purposes such as disinfection of hospital medical utensils, detoxifying textile wastes, and inactivation of algal bloom in large water bodies.

## Author contributions

CR, BJ, SR, GG, and RP wrote the manuscript. CR and BJ equally contributed to this article.

### Conflict of interest statement

The authors declare that the research was conducted in the absence of any commercial or financial relationships that could be construed as a potential conflict of interest.
